# Assessing the Tsetse Fly Microbiome Composition and the Potential Association of Some Bacteria Taxa with Trypanosome Establishment

**DOI:** 10.3390/microorganisms10061141

**Published:** 2022-05-31

**Authors:** Calmes Ursain Bouaka Tsakeng, Tito Tresor Melachio Tanekou, Steve Feudjio Soffack, Inaki Tirados, Cedrique Noutchih, Flobert Njiokou, Jude Daiga Bigoga, Charles Sinclair Wondji

**Affiliations:** 1 Centre for Research in Infectious Diseases (CRID), Yaoundé P.O. Box 13591, Cameroon; calmes.bouaka@crid-cam.net (C.U.B.T.); cedrique.noutchih@crid-cam.net (C.N.); njiokouf@yahoo.com (F.N.); charles.wondji@lstmed.ac.uk (C.S.W.); 2 Department of Biochemistry, Faculty of Science, University of Yaoundé I, Yaoundé P.O. Box 812, Cameroon; judebigoga@yahoo.com; 3 Department of Biological Sciences, Faculty of Science, University of Bamenda, Bamenda P.O. Box 39, Cameroon; 4 Department of Animal Biology and Physiology, Faculty of Science, University of Yaoundé I, Yaoundé P.O. Box 812, Cameroon; feudjiosteve36@yahoo.com; 5 Department of Vector Biology, Liverpool School of Tropical Medicine Pembroke Place, Liverpool L3 5QA, UK; inaki.tirados@lstmed.ac.uk

**Keywords:** tsetse flies, trypanosomes, microbiome, vector competence, vector control

## Abstract

The tsetse flies, biological vectors of African trypanosomes, harbour a variety of bacteria involved in their vector competence that may help in developing novel vector control tools. This study provides an inventory of tsetse bacterial communities in Cameroon and explores their possible associations with trypanosome establishment in *Glossina palpalis palpalis*. High throughput sequencing of the V3-V4 hypervariable region of the bacterial 16S rRNA gene, with subsequent metagenomic, multivariate, and association analyses, were used to investigate the levels and patterns of microbial diversity in four tsetse species. Overall, 31 bacterial genera and four phyla were identified. The primary symbiont *Wigglesworthia* dominated almost all the samples, with an overall relative abundance of 47.29%, and seemed to be replaced by *Serratia* or *Burkholderia* in some *G. tachinoides* flies. Globally, significant differences were observed in the microbiome diversity and composition among tsetse species and between teneral and non-teneral flies, or between flies displaying or not displaying mature trypanosome infections. In addition, differential abundance testing showed some OTUs, or some bacteria taxa, associated with trypanosome maturation in tsetse flies. These bacteria could be further investigated for an understanding of their mechanism of action and alternatively, transformed and used to block trypanosome development in tsetse flies.

## 1. Introduction

Human African Trypanosomiasis (HAT), or sleeping sickness, and Animal African Trypanosomiasis (AAT) are caused by protozoan parasites of the genus *Trypanosoma*, transmitted to vertebrates through the bite of infected tsetse flies (*Glossina* spp.). About 70 million people, 55 million cattle, and 70 million small ruminants are at risk of these diseases in 36 countries of sub-Saharan Africa [1,2]. AAT remains one of the major constraints to agriculture and livestock development. The economic losses resulting from the negative impact of this disease were estimated to be higher than USD 4.5 billion per year [3], and less than 1000 HAT cases now reported per year in Africa [4]. 

So far, there is no vaccine against trypanosomiasis, mainly due to the ability of trypanosomes to continually alter their surface glycoprotein layer through expressing distinct antigenic variants for immune evasion [5]. Currently, control of HAT relies essentially on the active detection and treatment of cases, which often reaches less than 75% of the affected population [6]. Moreover, the use of chemotherapy is limited by chemotoxicity and increasing levels of resistance to the available drugs [7,8]. Nevertheless, some efforts are being done to overcome this challenge with new therapies like nifurtimox-eflornithine combination treatment and the recent fexinidazole. 

Vector control intervention strategies with aim of reducing the tsetse fly population density and/or their ability to transmit trypanosomes are better complementary methods to help curbing the disease transmission. Indeed, to be transmitted, trypanosomes must first establish themselves in the tsetse fly midgut following a blood meal, and thereafter, mature in salivary glands or mouth parts, depending on the species [9]. This phenomenon is influenced by several factors, among which is the microbiome harboured by the tsetse vector, which are known to play diverse roles in their hosts. Three major symbionts are described in tsetse flies: the intracellular primary symbiont *Wigglesworthia glossinidia*, necessary for the fly’s fertility and immune response [10]; *Wolbachia* sp., which acts on the reproductive process of tsetse flies by inducing cytoplasmic incompatibility [11], and the secondary symbiont *Sodalis glossinidius*, present in gut and other tissues of the fly [12]. The latter was found to be involved in trypanosome establishment in the fly midgut through a complex biochemical mechanism involving the production of N-acetyl glucosamine [13], resulting from hydrolysis of pupae chitin to produce endochitinases. These molecules are known to inhibit a tsetse-midgut lectin, lethal to procyclic trypanosomes [14,15]. Beyond these three major symbionts, *Kosakonia cowanii* impairs trypanosome establishment in tsetse, while protecting this vector from the entomopathogenic *Serratia marcescens* [16]. 

Recent studies have shown a great diversity in the bacterial flora of *Glossina species* in different sleeping sickness foci in Cameroon [17,18,19,20,21] However, in these descriptive studies, no association was found between the presence of *S. glossinidius* and trypanosome infection in flies, suggesting that vector competence might rather be linked to given genotypes or the abundance of the symbiont [13]. Although previous studies that described the bacterial communities in tsetse flies have mainly focused on the midgut compartment [17,22,23], other insect tissues could harbour some bacteria taxa not yet described in the tsetse, or could be a key localization for other important bacteria detected in the gut. Therefore, studies are needed to make a complete inventory of the microbial community associated with tsetse flies in order to characterize and provide a more comprehensive overview of their composition and association with trypanosome establishment.

In the last decade, many studies on microbiome showed interesting results in developing alternative methods of fighting vector borne diseases, such as impairing parasite development in vectors and thus reducing disease transmission. Some microbes were used to shorten the insect vectors’ lifespan or to decrease their infection rates, either via natural competition mechanisms or via the production of genetically introduced anti-parasite molecules [24,25].

In the present study, we update the microbiome composition of *Glossina palpalis palpalis*, the main vector of HAT and AAT in the forest area of southern Cameroon, and we provide some information on the microbiome composition of three additional tsetse fly species in Cameroon.

## 2. Materials and Methods

### 2.1. Study Area

**Figure 1:**
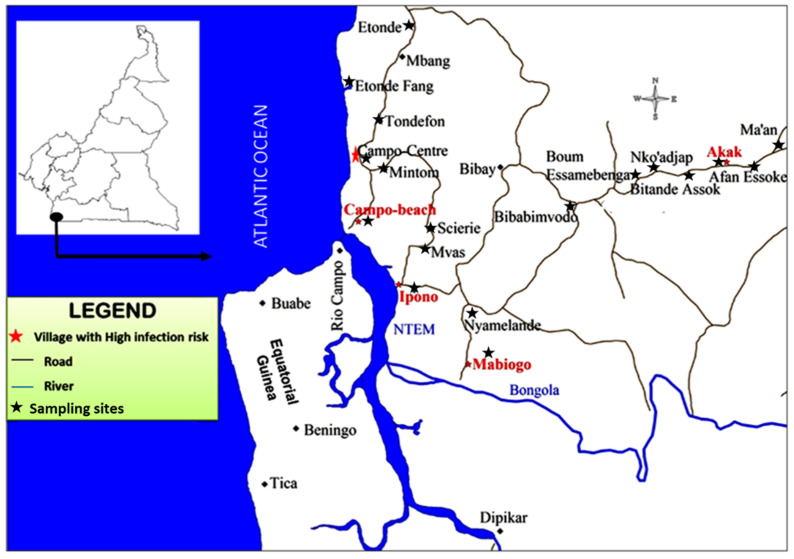
Map of Campo HAT focus (extracted and modified from the National Institute of Cartography Yaoundé Cameroon, 1976 topographic map).

Campo (2°20′ N, 9°52′ E) is located on the Atlantic coast, at the border with Equatorial Guinea (Figure 1) and extends along the River Ntem, where several cases of sleeping sickness are diagnosed every year. The climate is equatorial, with four seasons: the heavy and light rainy seasons (September to November and March to May, respectively) and the heavy and light dry seasons (December to February and June to August, respectively). The hydrographic network is dense and provides suitable areas for the development of tsetse flies, such as rivers, swampy areas, marshes, and mangrove forests. The Campo inhabitants are currently exposed to tsetse fly bites during activities such as fishing, hunting, and farming. In this region, wild fauna composition is highly diversified [26], and since 1932, it has been established as a wild fauna reserve. Several tsetse fly species (including *Glossina palpalis palpalis*, and to a lesser extent, *G. pallicera*, *G. caliginea*, and *G. nigrofusca*) were found in this study [27,28]. The study was conducted in almost all the villages in Campo (Afan Essoke, Akak, Assok, Campo-Beach, Campo-Centre, Essamebenga, Ipono, Etonde, Maan, Mabiogo, Mintomb, Mvass, Nyamelande, Nko’adjap, Okanbiloun, Tondefon, Etonde Fang, Scierie, Bibabimvodo) and on the banks of the River Ntem.

### 2.2. Tsetse Collection and Preservation

Tsetse flies were caught during the peak dry season, from 21 November to 12 December 2018, using pyramidal traps [29]. Traps were set up in various tsetse fly favourable biotopes (at water points, on riverbanks, behind dwellings, along the roads, in farmlands, etc.), and the geographical coordinates of each trap were recorded with a global positioning system. The flies were collected once a day during three consecutive days between 12 pm and 2 pm. The species, sex, and teneral status (i.e., if the fly is newly emerged and still unfed) of each collected tsetse fly were morphologically identified. Tsetse flies were then sterilized twice with 5% sodium hypochlorite and rinsed twice with distilled water to eliminate potential contaminants from the environment, as recommended in previous similar studies [17,21]. The head and legs of the flies were then separated from the rest of the bodies and the different parts conserved separately in well labelled microtubes containing ethanol 95°, for the determination of mature trypanosome infections and tsetse genetic population structure studies, respectively. Once in the laboratory, these samples were stored at −20 °C until DNA extraction.

### 2.3. DNA Extraction

DNA was extracted from fly heads and the rest of their bodies separately, using the LIVAK protocol [30]. Briefly, the alcohol used to preserve the head and the rest of the body of each fly was evaporated by maintaining the microtubes open overnight at room temperature (25 °C). Thereafter, each head and the rest of the body were crushed with pestles in 500 μL of LIVAK buffer (LIVAK: 1.6 mL NaCl 5 M; 5.48 g sucrose; 1.57 g Tris; 10.16 mL EDTA 0.5 M; 2.5 mL 20% SDS; distilled water to 100 mL total volume). The disrupted tissues were incubated at 65 °C for 30 min, and the aqueous upper phase containing the nucleic acids was obtained by adding 70 μL of potassium acetate, incubating in ice for 30 min, and centrifuging at 13,500 rpm for 20 min. DNA was precipitated by the addition of 1 mL of absolute ethanol and a centrifugation at 13,500 rpm for 15 min. DNA pellets were washed twice with 200 μL of 70% ethanol, centrifuged, and then dried at room temperature (25 °C) for 1 h. DNA pellets were finally re-suspended in 30 μL and 100 μL of sterile water for heads and bodies, respectively, before their storage at −20 °C for subsequent molecular analyses.

### 2.4. PCR Detection of Trypanosomes

Trypanosomes were detected using PCR-based methods from DNA extracts from tsetse bodies, and heads in the case that bodies were positive for at least one trypanosome species.

Trypanosomes’ ITS1 DNA was amplified by a nested ITS PCR, as described by Desquesnes et al. [31]. The primers used were TRYP18.2C (5′-GCAAATTGCCCAATGTCG-3′) and TRYP4R (5′-GCTGCGTTCTTCAACGAA-3′) for the first ITS-PCR and IRFCC (5′-CCTGCAGCTGGATCAT-3′) and TRYP5RCG (5′-ATCGCGACACCTTGTG-3′) for the second ITS-PCR. The amplification reactions were performed in a total volume of 20 μL containing 2 μL of TBE buffer 10X (10 mM Tris–HCl; 1.5 mM MgCl_2_, 50 mM KCl, pH 8.3), 0.56 μL of each primer (10 μM), 0.4 μL of dNTPs (10 mM), 0.2 μL of Taq DNA polymerase (5 U/μL), 14.28 μL H_2_O, and 2 μL of DNA extract for the first PCR; 2 μL of 1/10 diluted products of the first PCR for the second ITS-PCR. For both PCRs, the amplification programs involved an initial denaturation step at 94 °C for 5 min, followed by 30 amplification cycles. Each of these cycles included a denaturation at 94 °C for 30 s, an annealing step at 58 °C for 1 min, and extension at 72 °C for 1 min. The final extension step was performed at 72 °C for 5 min. The products from the second PCR were resolved on 2% agarose gel containing Midori green and visualized under UV light. 

### 2.5. Determination of Flies’ Microbiome Composition

#### 2.5.1. Library Preparation and Sequencing

Sequencing was performed with DNA extracted from 192 individual flies, using the Illumina MiSeq system (Polo d’ Innovazione di Genomica Genetica e Biologia, Siena, Italy). In this protocol, two specific primers targeting the V3-V4 region of the bacteria 16S rRNA gene, and flanked with the Illumina overhang adapters were used for the sequencing; namely, V3F: 5′-TCGTCGGCAGCGTCAGATGTGTATAAGAGACAGCCTACGGGNGGCWGCAG-3′ and V4R: 5′-GTCTCGTGGGCTCGGAGATGTGTATAAGAGACAGGACTACHVGGGT ATCTAATCC-3′ [32]. 

Amplicons were generated using a 2X KAPA HiFi HotStart Ready mix (KAPA Biosystems), through PCR reactions performed in a total volume of 25 μL, containing 2.5 μL DNA (5 ng/μL), 5 μL of each primer (1 μM), and 12.5 μL 2X KAPA HiFi HotStart Ready mix. The amplification program consisted of an initial denaturation step at 94 °C for 3 min, followed by 25 amplification cycles, each consisting of denaturation at 94 °C for 30 s, primer hybridization at 55 °C for 30 s, elongation at 72 °C for 30 s and final elongation at 72 °C for 5 min. The expected sizes of the PCR products were verified by running 1 μL of the PCR product on a bioanalyzer (expected size ~550 bp). PCR products were then cleaned up using AMPure XP beads (Beckman Coulter Genomics) to remove free primers and primer dimers. Then, 5 μL of purified products were used in the second PCR round to attach dual multiplexing indices (i5 and i7) and Illumina sequencing adapters using the Nextera XT Index Kit (Illumina catalogue), as recommended by the manufacturer. After this step, a second clean-up was performed using AMPure XP beads, and 1 μL of a 1:50 dilution of each sample was analysed on a bioanalyzer to verify the final size (~630 bp). Libraries were then normalized, and 5 μL of each were pooled. Finally, pooled libraries were denatured with NaOH diluted with hybridization buffer, heat-denatured, and loaded on the Illumina MiSeq flow-cell. Each run included 5% PhiX solution (Illumina catalogue) to serve as an internal control [33].

#### 2.5.2. Sequence Data Processing

Illumina MiSeq reads were analysed using Mothur v.1.44.3 [34], following a modified pipeline previously described for the same kind of analyses [35]. Briefly, forward and reverse demultiplexed paired-end reads were merged to contiguous sequences for each individual fly, and primers were trimmed, followed by quality filtering that removed all merged reads containing ambiguous bases. The dataset was reduced to unique sequences and a count-file summarizing the number of sequences of each type for all the flies. The unique sequences were then aligned to the V3-V4 region of the 16S rRNA gene sequences from the SILVA v.123 reference database and filtered to remove the overhangs at both ends. Then, the dataset was filtered to eliminate unique sequences with an abundance lower than 0.01%, probably issued from sequencing errors, and for the remaining sequences, highly similar samples (up to 1 difference each 100 base pairs) were pre-clustered for further denoising of the data. Another filtering step was performed to remove chimeric sequences, as well as those classified as eukaryote or mitochondria (probably from fly DNA), chloroplast, or unknown after alignment in the SILVA database. A distance matrix was generated between remaining clean sequences, and these were later clustered and classified into operational taxonomic units (OTUs). Finally, using the count-file, an OUT table was generated containing each individual fly, with all the OTUs it harbours, as well as their abundances.

#### 2.5.3. Statistical Analyses

Statistical analyses and plots were performed in the R environment [36]. Several packages were used for the different types or analyses performed: phyloseq [37] and microbiome [38] for the exploration and analysis of 16s microbiome data, taxonomic profiling, and association tests; ggplot2 [39] and ggpubr [40] for graphical plots visualization; vegan [41] for communities ecology (diversity analysis, community ordination, and dissimilarity analysis); DESeq2 [42] for differential OTU abundance testing between groups; ape [43] and dendextend [44] for the analyses of phylogenetic trees and hierarchical clustering; knitr [45] for dynamic reports generation. Rarefaction curves were performed prior to comparative analyses to ensure the sequencing depth was sufficient to describe all present taxa in all individual flies. Alpha diversity metrics were estimated using the Shannon diversity index (H) and compared between groups (fly species, trypanosome infected or not, teneral or not) using the Wilcoxon signed rank test. Principal components analysis (PCoA), using the Bray–Curtis dissimilarity index and ordination plots, was performed to determine differences in bacterial communities across samples of different groups, and the differences were quantified using permutational multivariate analysis of variance (PERMANOVA). Finally, differential abundance testing was performed to search potential taxonomic groups that can serve as biomarkers associated with a specific condition, mainly fly infectivity with trypanosomes, or ability to carry mature trypanosome infections in mouthparts. All tests considered statistical significance threshold of 0.05.

## 3. Results

### 3.1. Tsetse Fly Collection and Trypanosome-Infection Status

A total of 1915 tsetse flies were caught in Campo, among which 1789 (93.3%) were *Glossina palpalis palpalis*, 73 (3.8%) *G. pallicera pallicera*, 47 (2.5%) *G. caliginea*, 5 (0.3%) *G. nigrofusca*, and one fly could not be identified morphologically. The teneral flies accounted for 5.1%. Among 1054 *G. palpalis palpalis*, 177 (16.79%) had their midguts infected by at least one trypanosome species, as revealed by PCR. The most frequent infection found was *Trypanosoma congolense* (15.37%), followed by *T. brucei* s. l. (1.52%), *T. vivax* (0.47%), and *T. simiea* (0.47%). Eleven flies (5.85%) were carrying a mixed infection, and of the 16 flies infected with *T. brucei* s. l., one was identified as *T. brucei gambiense*. Among the infected flies, 40 (21.27%) showed the presence of *T. congolense* DNA in the mouthparts, and these flies were considered as carrying a mature infection.

### 3.2. Bacterial 16S rRNA Sequencing Quality and Rarefaction

The V3-V4 hyper-variable region of the bacterial 16S rRNA gene was analysed in 192 individual field-collected tsetse flies, including 132 *Glossina palpalis palpalis* (72 were harbouring trypanosome infections in their midguts, of which 32 were carrying a mature infection), 40 were uninfected *G. palpalis palpalis* and 20 were teneral, 20 were *G. caliginea*, and 20 were *G. pallicera* (also captured in Campo), and 20 were *G. tachinoides* (from the Dodeo animal trypanosome focus in the Adamaoua Region, Cameroon).

A total of 96 million raw reads were obtained from the sequencing company, and after quality control and filtering, 24 million clean reads remained for subsequent analyses. Rarefaction curves reached a plateau at around 6000 reads, while the sample with the smallest number of reads had 40,000 reads, showing that the sequencing effort was sufficient to characterize most or all the taxa present.

### 3.3. General Characterization of Bacterial Phyla and Abundances in Tsetse

A total of 85 bacterial OTUs were detected in the 4 tsetse species examined. These bacterial OTUs belonged to 4 phyla and 31 genera (Supplementary Table S1). Most of the sequences were identified as belonging to the phylum Proteobacteria (95.04%) and were present in all the 192 samples. The relative abundance of other bacteria phyla described was 4.45% for *Firmicutes*, 0.30% for *Chlamydiae*, 0.08% for *Acidobacteria*, and 0.13% of sequences could not be classified in a particular phylum.

**Figure 2:**
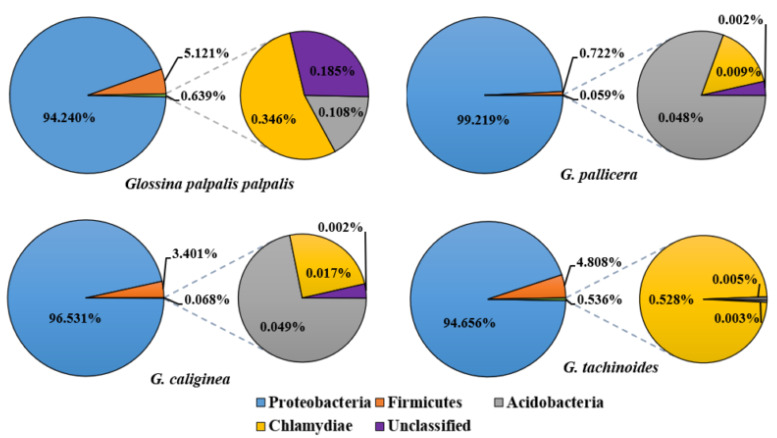
Relative abundance of bacterial phyla in tsetse flies.

The overall bacterial phyla identified were unevenly distributed in the different fly species, i.e., Proteobacteria represented 99.219% in *G. pallicera pallicera*, 96.531% in *Glossina caliginea*, 94.656% in *G. tachinoides*, and 94.240% in *G. palpalis palpalis* (Figure 2).

### 3.4. Tsetse Microbiome Composition at Genus Level

The most abundant bacteria genus was *Wigglesworthia*, the primary symbiont of tsetse flies with a relative abundance of 47.29%. The other abundant genera found were *Serratia* (16.58%), *Pantoae_Klebsiella_Enterobacter_Kluyvera* (16.30%), which were highly similar in their V3-V4 sequences and could not be distinguished, *Pseudomonas* (6.16%), *Staphylococcus* (3.39%), *Acinetobacter* (2.77%), *Stenotrophomonas* (2.03%), and *Burkholderia* (1.38%). The 4.1% remaining were shared by other less-represented genera (Table 1), including 0.93% being unclassified.

**Figure 3:**
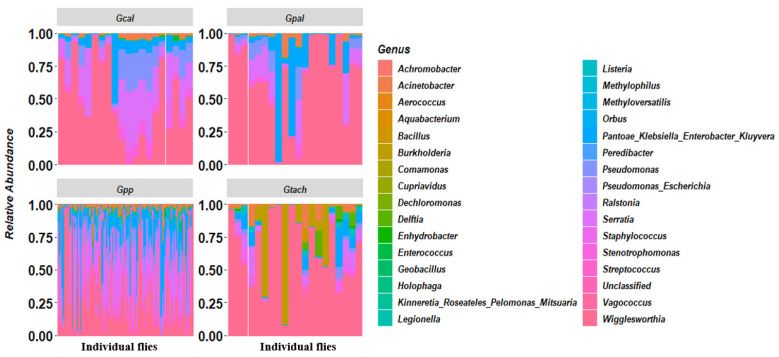
Map showing the relative abundance and distribution of the bacterial genera within different tsetse species (*Gtach*: *Glossina tachinoides*; *Gpp*: *G. palpalis palpalis*; *Gcal*: *G. caliginea*; *Gpal*: *G. pallicera*).

The bacteria genera described were also unevenly distributed among different tsetse species and individual tsetse flies. *Wigglesworthia* displayed an overall abundance of 65.64% in *G. pallicera pallicera*, 62.61% in *G. tachinoides*, 47.61% in *G. caliginea*, and 42.13% in *G. p. palpalis* (Figure 3, Supplementary Table S1). However, this bacterium seems to be replaced as the predominant symbiont by *Pantoae_Klebsiella_Enterobacter_Kluyvera* in some *G. palpalis palpalis* samples, as T19n12 (96.08%), T19n16 (95.61%), T18n2 (94.95%), and T11n5 (84.3%), or by *Burkholderia* in some *G. tachinoides* samples, including M17 (91.9%), M14 (69.9%), and M4 (47.18%), or by *Serratia* in *G. palpalis palpalis* in samples T40n2 (54.2%), T19n38 (42.21%), and T31n1 (40.3%) (Supplementary Table S2).

Globally, concerning the distribution of other abundant bacteria in tsetse fly species, *Serratia* was the second most abundant bacterium in *G. caliginea* (21.7%) followed by *Pseudomonas* (11.95%), *Pantoae_Klebsiella_Enterobacter_Kluyvera* (8.10%), and *Staphylococcus* (2.96%). However, in *G. pallicera pallicera*, *Pantoae_Klebsiella_Enterobacter_Kluyvera* (14.81%) was the second most abundant bacterium, followed by Serratia (8.95%), *Pseudomonas* (4.80%), and *Acinetobacter* (2.35%). In *G. palpalis palpalis*, the trend was similar to *G. pallicera pallicera*, with *Pantoae_Klebsiella_Enterobacter_Kluyvera* (19.45%) in second, followed by *Serratia* (18.82%) and *Pseudomonas* (6.16%); However, here, *Acinetobacter* was replaced by *Staphyloccocus* (3.96%). Finally, in *G. tachinoides*, *Burkholdderia*, while completely absent in *G. pallicera* and very less represented in *G. palpalis palpalis* and *G. tachioides*, was the second most abundant bacteria (13.02%) followed by *Pantoae_ Klebsiella_ Enterobacter_ Kluyvera* (5.18%), *Serratia* (4.3%), and *Acinetobacter* (3.62%). Some bacteria, such as *Cupriavidus*, were only present in *G. palpalis palpalis*, while *Orbus*, *Vagococcus*, and *Dechloromonas* were only present in *G. palpalis palpalis* and *G. pallicera pallicera*.

### 3.5. Bacterial Genera Diversity in Tsetse Flies (Alpha-Diversity)

#### 3.5.1. Diversity in Tsetse Species

**Figure 4:**
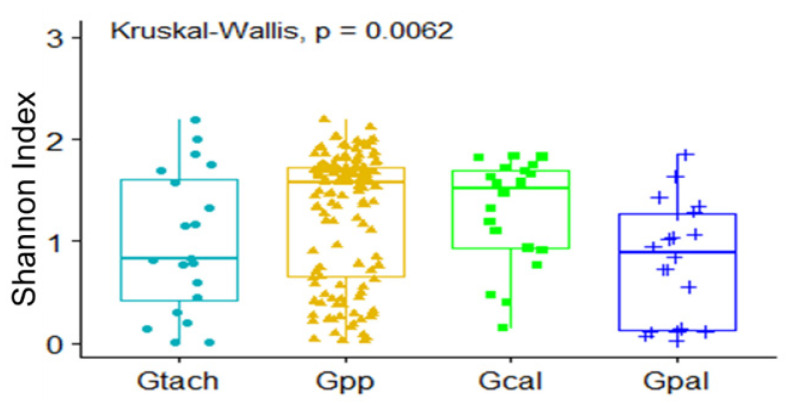
Microbiome diversity in different tsetse species (Gtach: *Glossina tachinoides*; Gpp: *G. palpalis palpalis*; Gcal: *G. caliginea*; Gpal: *G. pallicera pallicera*).

Bacterial genera richness and evenness were significantly different between tsetse species (Figure 4; *p*-value = 0.0062). Briefly, the gut microbiota of *G. p. palpalis* was significantly richer in genera compared to that of *G. pallicera* (*p*-value = 0.0072). However, no significant difference was observed when comparing *G. palpalis palpalis* and *G. caliginea* (*p*-value = 0.9554), *G. tachinoides* and *G. caliginea* (*p*-value = 0.9554), *G. tachinoides* and *G. pallicera* (*p*-value = 0.4960), and *G. tachinoides* and *G. palpalis palpalis* (*p*-value = 0.3792).

#### 3.5.2. Diversity in Non-Teneral and Teneral *Glossina palpalis palpalis*

**Figure 5:**
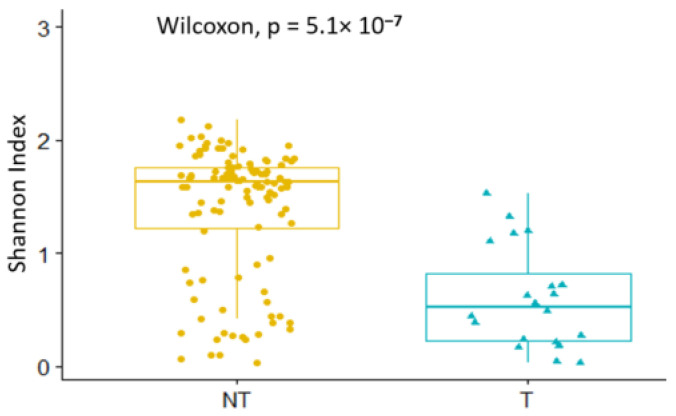
Microbiome diversity in non-teneral and teneral flies (T: teneral; NT: non-teneral).

In non-teneral tsetse flies of the species *G. palpalis palpalis*, the top represented bacteria were *Wigglesworthia* (37.10%), *Serratia* (20.97%), *Pantoae_Klebsiella_ Enterobacter_Kluyvera* (20.51%), *Pseudomonas* (6.99%), and *Staphyloccocus* (4.44%), while teneral flies were dominated by *Wigglesworthia* (70.30%), *Pantoae_Klebsiella_Enterobacter_Kluyvera* (13.52%), *Serratia* (6.79%), *Acinetobacter* (3.21%), and *Pseudomonas* (1.52%). *Orbus* and *Burkholderia* were found only in non-teneral flies (Supplementary Table S3). In summary, the abundance of *Wigglesworthia* in flies decreased, whereas the abundance of most of other bacteria genera increased through the process of blood feeding, and the whole microbiome diversity of teneral flies was lower than that of non-teneral flies (*p*-value < 10^−4^) (Figure 5).

### 3.6. Multivariate Analysis (Beta Diversity)

**Figure 6:**
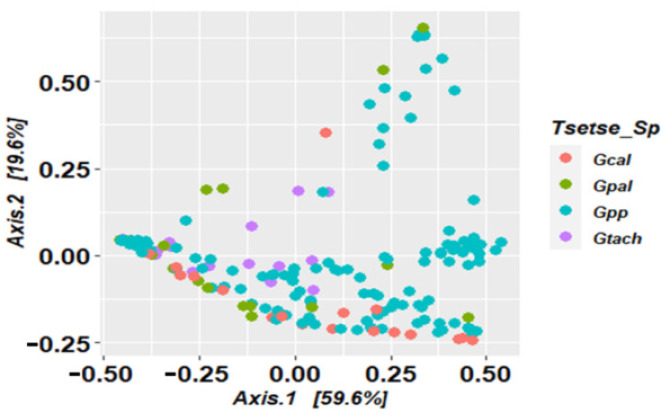
Distribution of the tsetse flies according to their microbiome composition, based on principal coordinates analysis using Bray–Curtis index (Gcal: *G. caliginea*; Gpal: *G. pallicera pallicera*; Gpp: *G. palpalis palpalis*; Gtach: *G. tachinoides*).

No clear separation or clustering of flies’ microbiome composition was observed according to their species, as shown by the principal coordinate analysis (PCoA) performed using the Bray–Curtis dissimilarity index (Figure 6). Nevertheless, the level of dissimilarity was significant after the permutational analysis of variance (PERMANOVA), showing a difference in the composition of the fly microbiota in different tsetse species (*p*-value = 0.01). The community structure and composition of the microbiota of *Glossina palpalis palpalis* was substantially different from that of *G. caliginea* (*p*-value = 0.048) and *G. pallicera pallicera* (*p*-value = 0.012). However, *G. caliginea*, *G. pallicera pallicera*, and *G. tachinoides* had a similar pattern in their microbiota when compared to each other (*p*-value values of 0.64; 0.94 and 0.70, respectively).

**Figure 7:**
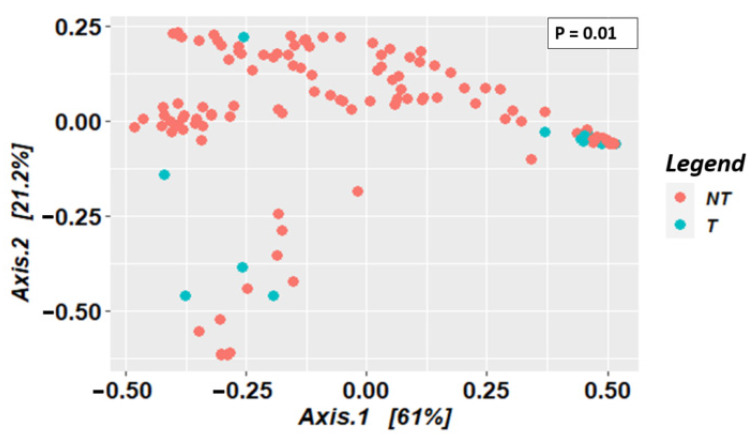
Distribution of tsetse fly samples according to their microbiota composition, based on principal coordinates analysis using the Bray–Curtis index, in non-teneral and teneral flies (NT: non-teneral; T: teneral).

In *Glossina palpalis palpalis*, although a great diversity was observed in the microbiome composition of non-teneral flies, most of the teneral ones clustered together on the PCoA plot, showing that they have a similar composition in the bacterial genera present, as well as their abundances (Figure 7). Moreover, a significant difference in beta diversity was observed between non-teneral and teneral tsetse flies (*p*-value = 0.01).

### 3.7. Bacterial Communities in Glossina palpalis palpalis Infected and Non-Infected Flies

**Figure 8:**
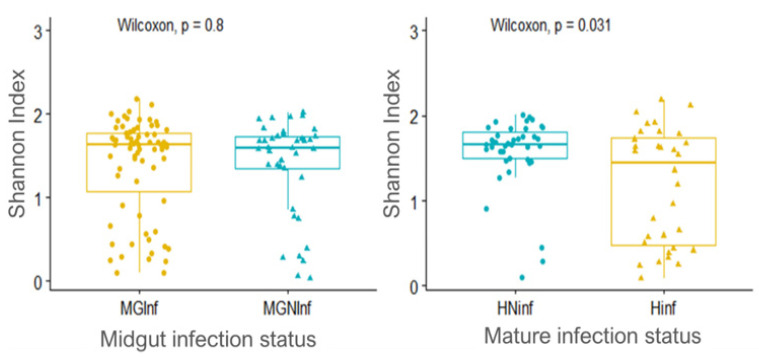
Tsetse microbiome alpha diversity regarding trypanosome infection status (MGInf: midgut infected; MGNInf: midgut non-infected; HNinf: head non-infected; Hinf: Head infected).

Globally, no statistically significant difference was observed when comparing bacteria richness and evenness between *G. palpalis palpalis* flies harbouring trypanosomes in their midgut and non-infected flies (Shannon index, *p*-value = 0.8), as shown in the Figure 8. However, within the infected tsetse flies, a significant difference was observed between samples harbouring a mature trypanosome infection and without infections in the mouthparts (Shannon index, *p*-value = 0.031). This result suggested a similar diversity for midgut infected and non-infected flies, that seemed to change when the infection become mature.

**Figure 9:**
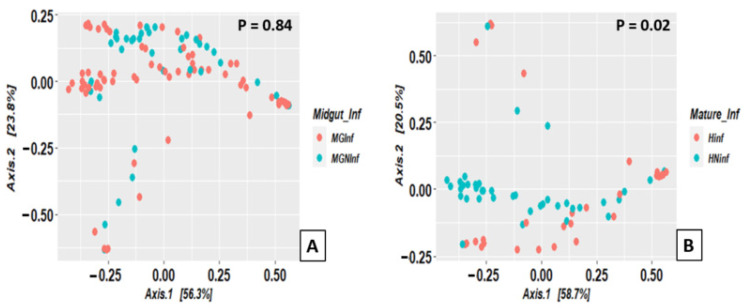
Distribution of tsetse fly samples according to their microbiome composition, based on principal coordinates analysis using the Bray–Curtis index, within trypanosome infection status: (**A**) midgut infection, (**B**) mature infection (MGInf: midgut infected; MGNInf: midgut non-infected; Hinf: head infected or mature infection; HNinf: head non-infected).

Regarding the beta diversity, the tsetse flies studied had similar microbiome composition (taxa richness and abundance) regardless of if they harboured trypanosome infections in their midguts or not, confirmed by the PERMANOVA test (*p*-value = 0.84). However, comparing the microbiome composition of the flies harbouring a mature trypanosome infection and those only infected in the midgut, a significant difference was observed (Figure 9, PERMANOVA *p*-value = 0.02).

**Figure 10:**
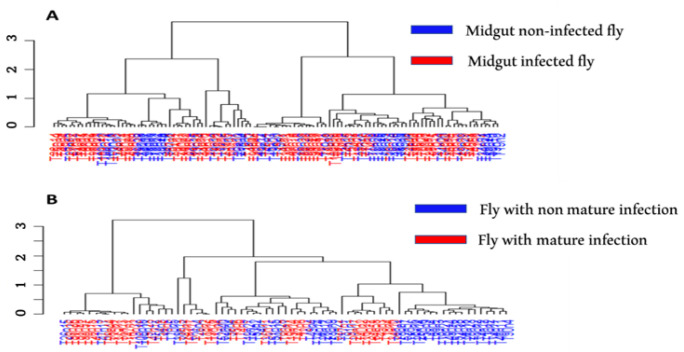
Hierarchical cluster dendrogram, based on Bray–Curtis Index values, showing the relationship between different tsetse microbiome communities and infection status. (**A**) midgut infection with trypanosomes, (**B**) mature infection with trypanosomes.

Furthermore, the hierarchical clustering using the Bray–Curtis dissimilarity index clearly showed a similarity in microbial communities of flies according to infection status (midgut infection and mature infection), despite the high variability observed in the different groups and no clear “higher-level” clustering of infected flies on one hand, and uninfected ones on the other hand (Figure 10).

Looking deeply at the difference in the diversity of the tsetse flies harbouring trypanosome infections in their midguts or mature and non-mature infections, differential abundance testing showed many OTUs presenting significantly different abundances, as summarized in the Table 2 and Table 3. Indeed, numerous genera and OTUs contributed to differences between samples with high fold change, the most important being *Dechloromonas*, *Ralstonia*, *Listeria, Kinneretia_Roseateles_Pelomonas_Mitsuaria*, *Enhydrobacter*, and *Staphylococcus*.

## 4. Discussion

The description of microbiota harboured by arthropod vectors presents increasing interest, owing to their role in modulating vector fitness or competence and their potential use in vector control. Although most studies on the tsetse fly vector of human and animal trypanosomiases have mainly described the microbial diversity in tsetse fly guts, few have either looked at the microbiome in the whole fly or have established strong associations between the microbiome composition and the maturation of trypanosomes in the flies. In the present study, we conducted a high throughput sequencing of the V3–V4 region of the bacterial 16S rRNA gene in the tsetse fly *Glossina palpalis palpalis*, the main vector of human sleeping sickness in the forest area of Cameroon. We included a few samples of three additional tsetse species in order to make a complete inventory of the microbial communities they harbour. Bacterial taxa associated with the infection of tsetse with trypanosomes, or the maturation of these later, were also investigated.

### 4.1. Abundances of Bacterial Taxa in Whole Tsetse Fly Bodies

Using whole tsetse fly bodies, a total of 85 OTUs, belonging to 32 bacterial genera, were identified in 4 phyla. They were largely dominated by the phylum *Proteobacteria*, with a mean relative abundance of 95.04%. This observation is consistent with other earlier studies showing the predominance of this phylum (~90%) in the guts of tsetse flies [17,21,46]. Such predominance is due to the high relative abundance of the primary symbiont *Wigglesworthia* that represented 47.29% of the total microbiome and other taxa like *Serratia* (16.58%), *Pantoae_Klebsiella_Enterobacter_Kluyvera* (16.30%), and *Pseudomonas* (6.16%). This result is not surprising, since *Proteobacteria* species are known to easily adapt and develop in different biotopes. The high abundance of the primary mutualist symbiont of tsetse flies, *Wigglesworthia*, corroborates the result reported in previous studies [47]. However, the relatively lower abundance of *Wigglesworthia* found in the present study (47% of the tsetse gut bacteriome) compared to previous studies (90–99%) is probably due to the fact that whole fly bodies were investigated here, rather than the midgut only. *Wigglesworthia* may be predominant in the midgut, whereas the other taxa may be more abundant in other tissues; this result suggest that the abundances of other bacteria taxa were largely underestimated in the previous studies, where midguts only were used for the analysis. Despite that *Wigglesworthia* was the predominant bacteria in most of the flies, some *Glossina palpalis palpalis* individuals were dominated by *Pantoae_Klebsiella_Enterobacter_Kluyvera* or *Serratia*, and some *G. tachinoides* were dominated by *Burkholderia*. Members of the genus *Burkholderia* are widespread in soil rhizospheres and plant surfaces, and some species are known to be associated with insects feeding on plants [48,49,50]. A previous study reported that tsetse flies may ingest bacteria present on the epidermis of a variety of vertebrates [27] or in plants’ nectar when they feed [51,52]. Although infection by *Burkholderia* is non-essential for growth and reproduction of the mosquito [53], for example, association studies revealed mutualistic relationships with insect, where the symbiont presence increases the insect fitness or protects the insect from entomopathogenic fungi [50,54]. On the other hand, *Pantoea* has been shown to cross-colonise several mosquito species and is readily transformed and cultured, and therefore, has been proposed for paratransgenic applications [55]. Further investigations on these predominant bacteria dynamics throughout the tsetse fly life cycle are required to better define the nature of the microbe–fly association. *Serratia* was detected in more than 90% of the flies in our study, with an overall abundance of 16.58%; this was not expected, since this bacterium has been previously reported in only around 50% of flies by Jacob et al. [17] and with only 0.0012% abundance by Tsagmo-Ngoune et al. [20]. This observation strengthens the hypothesis that fly tissues other than those in the midgut are key localizations for bacteria development, and the potential importance of this bacteria deserves to be further investigated.

### 4.2. General Microbiome Diversity in Tsetse Flies

Bacterial taxa richness and evenness were different between tsetse fly species. The microbiota composition of *G. palpalis palpalis* and *G. caliginea* were significantly more diverse and evenly distributed compared to that of *G. pallicera* and *G. tachinoides*. This result may be due to the differences in the environmental conditions of these different tsetse species and in the food supply from which some of these microbes originate, as suggested previously [23,56]. Moreover, differences in the gut physiological conditions and/or the fly’s innate immune system may impair the proliferation of some bacterial taxa in different fly species, modulating the composition of microbial communities, as suggested in studies on mosquitoes [57]. This observation concurs with the significant difference detected in the alpha and beta diversities when comparing non-teneral and teneral *G. palpalis palpalis*, indicating that blood meals have a significant impact on the tsetse microbiome. Indeed, an increase in the relative abundance of *Pantoae_Klebsiella_Enterobacter_Kluyvera*, *Serratia*, *Pseudomonas*, *Staphyloccocus*, *Methylophilus*, and other bacteria from teneral to non-teneral flies was concomitant with a decrease in *Wigglesworthia* from 70% in teneral flies, to 37% in non-teneral flies. A similar result was observed in blood-fed ticks compared to unfed ticks [58]. However, more work is needed to obtain a complete and accurate picture of the bacteria associated with blood meals of the tsetse fly and to understand how and why these bacteria establish in their hosts.

### 4.3. Microbiome of Trypanosome Infected and Non-Infected Glossina Palpalis Palpalis

Although no significant difference was observed when comparing bacteria richness and evenness between flies harbouring trypanosomes in their midguts and non-infected flies, the abundances of some bacteria taxa were nevertheless different in the two groups. This result is consistent with previous results obtained by Jacob et al. [17] and Tsagmo-ngoune et al. [20] on the same tsetse species in Cameroon. However, alpha and beta diversities of the tsetse microbiome were significantly different between flies harbouring mature trypanosome infections and those with only non-mature infection in the midgut. Indeed, a significant drop was observed in the alpha diversity of flies with mature trypanosome infection, and this observation was strengthened by hierarchical clustering of these flies, at least at low levels, distinguishing them from those with non-mature trypanosome infections. Differential abundance testing showed some bacteria phylotypes from the genera *Dechloromonas*, *Ralstonia*, *Serratia*, *Pseudomonas*, *Enteroccocus*, *Wigglesworthia*, *Methylophilus*, *Escherichia*, *Enhydrobacter*, and *Staphyloccocus* associated with the infection status of flies. The roles of these bacteria genera in tsetse fly biology remain unknown and poorly documented in other insects. *Enterobacter*, *Escherichia coli*, *Serratia marcescens*, and *Enterococcus* spp. are able to produce toxic molecules with potential antiparasitic activity (such as prodigiosin [59]) that were shown to be toxic to *Plasmodium falciparum* [60] and to *Trypanosoma cruzi* [61]. However, *Serratia odorifera* was shown to enhance the susceptibility of *Aedes aegypti* to the chikungunya virus [62], as well as its susceptibility to the dengue-2 virus [63]. In addition, the genus *Bacillus* is thought to be essential for *Culex pipiens* reproduction [64], and some species were shown to play a role in the digestion of polysaccharides and aromatic compounds such as chitin and lignocellulose in termites [65]. *Pseudomonas aeruginosa* are known to play an important role in mosquito *Culex quinquefasciatus* and *Culex tarsalis* adaptation to hypereutrophic aquatic habitats [66]. Therefore, based on the importance of different bacteria taxa in other insects, further investigations into their potential roles in tsetse fly biology, or their interaction with the trypanosomes that these flies transmit, are required. Moreover, the identification of *Wigglesworthia* OTU 18 and *Serratia* OTU 55 associated with the tsetse fly infection, rather than the predominant OTUs (OTU 01 and OTU 03, respectively, for the two genera), further justifies the fact that vector competence might be linked to given bacterial genotypes or their abundance, as previously suggested by Geiger et al. [13], rather than simply the presence/absence of the bacteria taxa.

The endosymbionts *Sodalis glossinidius* and *Wolbachia*, commonly reported in tsetse flies [17,56,67], could not be described in this study, since they were eliminated by the abundance threshold of 0.01% set for the analyses. These symbionts are known to be involved in modulating the ability of the tsetse fly to acquire trypanosomes [68,69] and to induce a variety of reproductive phenotypes, such as cytoplasmic incompatibility, parthenogenesis, and feminization, into the host population, respectively [70,71]. As these symbionts exhibit a wide tissue tropism and can be found intra or extra-cellularly in various tissues, including the midgut, fat body, milk gland, salivary glands and hemocoel [12,72], it is probable that they have a generally low relative abundance, which is a constraint to their identification and thus, a limitation to the NGS method used.

## 5. Conclusions

The present study provides some updates on the composition and diversity of the tsetse fly microbiota in Cameroon. Of the 192 individual tsetse flies, the metagenomic analysis performed resulted in the detection of 85 OTUs belonging to 31 bacterial genera and 4 phyla. The current study, using the whole fly body, revealed a higher bacterial diversity than previously observed in the midgut only, which indicates that various localizations other than the midguts should be considered in further investigations of the tsetse fly microbiome. Moreover, the significant difference in microbiome diversity between flies harbouring mature and non-mature trypanosome infections suggests either a change in microbiome diversity and composition for infection maturation, or that trypanosome maturation is possible only with the particular abundances of some microbial taxa. Therefore, the strong association between some identified bacteria genera and trypanosome infection status deserves additional investigations for the development of novel tools to control disease transmission by blocking the trypanosome development in tsetse flies.

## Figures and Tables

**Table 1:** Summary of bacterial genera abundance according to the flies’ species.

Genus	*G. cal.* %	*G. pal.* %	*G. p. p.* %	*G. tach* %	Total Abundance (%)
***Wigglesworthia***	47.61	65.64	42.13	62.61	47.29
***Serratia***	21.70	8.95	18.82	4.33	16.58
***Pantoae_Klebsiella_*** ***Enterobacter_Kluyvera***	8.10	14.81	19.45	5.18	16.30
***Pseudomonas***	11.95	4.80	6.16	1.69	6.16
***Staphylococcus***	2.96	0.69	3.96	2.43	3.36
***Acinetobacter***	1.78	2.35	2.85	3.62	2.77
***Stenotrophomonas***	3.97	1.58	2.06	0.39	2.03
***Burkholderia***	0.01	0	0.03	13.02	1.38
**Others**	1.87	1.14	4.49	6.69	4.10

Notes: *G. cal.*: *Glossina caliginea*; *G. pal.*: *G. pallicera pallicera*; *G. p. p.*: *G. palpalis palpalis*; *G. tach*: *G. tachinoides*.

**Table 2:** Operational Taxonomic Units displaying a significant difference in abundance between tsetse flies with midguts non-infected vs. infected with trypanosomes.

OTUs	Genus (Percentage/Num OTUs in the Genus) *	Base Mean	log2 Fold Change	lfcSE	Statistic	*p*-Value
**OTU37**	*Dechloromonas* (100/1)	21.44	−25.44	2.3	−11.06	0.0000
**OTU49**	*Ralstonia* (100/1)	19.88	−10.58	1.67	−6.33	0.0000
**OTU14**	*Listeria* (100/1)	283.11	−5.41	1.08	−5.01	0.0000
**OTU28**	*Aquabacterium* (79.12/2)	46.11	−7.76	1.99	−3.9	0.0001
**OTU25**	*Bacillus* (53.62/3)	38	−2.98	0.9	−3.31	0.0009
**OTU24**	*Methylophilus* (24.65/2)	11.6	−6.3	2.25	−2.8	0.0051
**OTU70**	*Kinneretia_ Roseateles_ Pelomonas_Mitsuaria* (100/1)	11.36	−4.37	1.59	−2.74	0.0061
**OTU33**	*Pseudomonas_ Escherichia* (100/1)	9.31	−2.98	1.19	−2.51	0.0120
**OTU45**	*Pseudomonas* (0.36/3)	4.66	−5	2.11	−2.37	0.0179
**OTU63**	*Cupriavidus* (40.19/2)	3.25	−4.82	2.18	−2.21	0.0273
**OTU27**	*Enterococcus* (70.41/3)	5.99	−5.36	2.52	−2.13	0.0334
**OTU18**	*Wigglesworthia* (0.25/2)	15.97	4.57	0.84	5.43	0.0000
**OTU55**	*Serratia* (0.14/2)	10.16	3.39	0.96	3.51	0.0004
**OTU40**	*Staphylococcus* (1.85/7)	44.63	5.48	1.89	2.9	0.0037

* Percentage of the OTU/number of OTUs for the genus concerned; lfcSE: log2 fold change standard error.

**Table 3:** Operational Taxonomic Units displaying a significant difference in abundance between tsetse flies with non-mature vs. mature infection with trypanosomes.

OTUs	Genus (Percentage/Num OTUs in the Genus) *	Base Mean	log2 Fold Change	lfcSE	Statistic	*p*-Value
**OTU29**	*Staphylococcus* (6.19/7)	7.85	−24.84	2.92	-8.51	0.0000
**OTU55**	*Serratia* (0.14/2)	1.59	−3.02	1.32	-2.28	0.0224
**OTU49**	*Ralstonia* (100/1)	21.57	−3.88	1.77	-2.19	0.0286
**OTU16**	*Enhydrobacter* (100/1)	238.65	2.66	0.84	3.15	0.0016
**OTU25**	*Bacillus* (53.62/3)	37.31	2.35	1.07	2.19	0.0287

* Percentage of the OTU/number of OTUs for the genus concerned; lfcSE: log2 fold change standard error.

## Data Availability

All data generated or analysed during this study are included within the article and its additional files. The sequences generated have been deposited in the GenBank database (study accession number: Primary-PRJNA811809; Secondary-PRJNA812727).

## Supplementary Materials

The following supporting information can be downloaded at: https://www.mdpi.com/article/10.3390/microorganisms10061141/s1, Table S1: Summary of bacterial abundances in different individual tsetse samples; Table S2: Bacterial genera abundance according to the flies’ species; Table S3: Summary of bacterial genera abundance in different groups of tsetse flies.
