# Supplementary material for: Assessing the Tsetse Fly Microbiome Composition and the Potential Association of Some Bacteria Taxa with Trypanosome Establishment
Source: Microorganisms. 2022 May 31;10(6):1141. doi: 10.3390/microorganisms10061141 (PMC9229743; doi:10.3390/microorganisms10061141)
Supplement: Supplementary file 1 [file microorganisms-10-01141-s001.zip › microorganisms-1731762-supplementary-2/Suplementary Table S2_Bouka et al.pdf]

Supplementary Table S2: Bacterial genera abundance according to the flies' species

Genus	*G. cal.* %	*G. pal.* %	*G. p. p.* %	*G. tach* %	Total abundance (%)
Wigglesworthia	47.610182	65.642191	42.137893	62.611812	47.2936772
Serratia	21.702030	8.951618	18.823373	4.332136	16.5834137
Pantoae_Klebsiella_ Enterobacter_Kluyvera	8.108133	14.817094	19.456272	5.183636	16.3013454
Pseudomonas	11.958733	4.808630	6.165262	1.699745	6.1622939
Staphylococcus	2.969413	0.694511	3.968475	2.432323	3.3627958
Acinetobacter	1.780862	2.354777	2.856591	3.629718	2.7727156
Stenotrophomonas	3.976580	1.581399	2.064466	0.396511	2.0395646
Burkholderia	0.015110	0	0.031788	13.022083	1.3810743
Unclassified	0.564854	0.692730	1.012066	1.049295	0.9360245
Listeria	0.031633	0.017813	0.469581	1.554900	0.4899692
Methylophilus	0.028071	0.002641	0.643010	0.078872	0.4533113
Delftia	0.226040	0.112650	0.105101	2.418686	0.3597069
Orbus	0.000123	0	0.410778	0	0.2823053
Enhydrobacter	0.042137	0.014680	0.276657	0.341165	0.2316157
Achromobacter	0.416761	0.160007	0.173112	0.001167	0.1792226
Bacillus	0.002641	0.003378	0.160923	0.623914	0.1762639
Aquabacterium	0.009828	0.005282	0.216017	0.001536	0.1501858
Vagococcus	0.001106	0	0.193381	0	0.1330097
Enterococcus	0.388444	0.000369	0.112715	0.003256	0.1183375
Geobacillus	0.001966	0.000921	0.141567	0.000123	0.0976006
Legionella	0.006081	0.007248	0.075420	0.248103	0.0790860
Holophaga	0.047296	0.045392	0.075597	0	0.0616152
Streptococcus	0.005160	0.003378	0.055384	0.184772	0.0582145
Cupriavidus	0	0	0.082474	0	0.0566775
Dechloromonas	0.001106	0	0.071245	0	0.0490757
Pseudomonas_Escherichia	0.094654	0.070268	0.037836	0.000123	0.0432094
Ralstonia	0.000553	0.000061	0.045990	0.075862	0.0395782
Methyloversatilis	0.005344	0.004730	0.030995	0.096747	0.0324375
Comamonas	0.004914	0.006572	0.031033	0	0.0225237
Kinneretia_Roseateles_ Pelomonas_Mitsuaria	0	0.000061	0.030725	0	0.0211212
Peredibacter	0.000061	0	0.025283	0.004361	0.0178358
Aerococcus	0.000184	0.001597	0.018992	0.009153	0.0141918

G. cal.: *Glossina caliginea*; *G. pal.*: *G. pallicera pallicera*; *G. p. p.*: *G. palpalis palpalis*; *G. tach*: *G. tachinoides*
