# Supplementary material for: Assessing the Tsetse Fly Microbiome Composition and the Potential Association of Some Bacteria Taxa with Trypanosome Establishment
Source: Microorganisms. 2022 May 31;10(6):1141. doi: 10.3390/microorganisms10061141 (PMC9229743; doi:10.3390/microorganisms10061141)
Supplement: Supplementary file 1 [file microorganisms-10-01141-s001.zip › microorganisms-1731762-supplementary-2/Suplementary Table S3_Bouka et al.pdf]

Supplementary Table S3: Summary of bacterial genera abundance in different groups of tsetse flies

Genus	MGInf (%)	MGNInf (%)	HInf (%)	HNInf (%)	NT (%)	T (%)
**Wigglesworthia**	39.066157	50.020640	45.988990	34.865451	37.1085	70.3097
**Pantoae_Klebsiella_ Enterobacter_Kluyvera**	20.212214	13.473089	16.706950	22.421387	20.5158	13.5212
**Serratia**	19.548457	16.291457	17.547673	20.825939	20.9704	6.7966
**Pseudomonas**	6.417254	7.126770	6.178006	6.578372	6.9935	1.5258
**Staphylococcus**	3.226800	4.045775	2.961774	3.397441	4.4455	1.2962
**Acinetobacter**	3.025839	2.447747	3.150567	2.954917	2.7925	3.2154
**Stenotrophomonas**	2.288195	2.302296	1.868167	2.552487	2.4313	0.0098
**Unclassified**	1.180123	0.691701	1.193990	1.149876	1.0228	0.9521
**Listeria**	0.773025	0.230305	0.592645	0.886124	0.5533	0.0004
**Orbus**	0.645767	0.000030	0.000083	0.935185	0.4841	0
**Burkholderia**	0.592105	1.691628	0.011642	0.952248	0.0375	0
**Methylophilus**	0.560750	0.166929	0.841759	0.388134	0.5307	1.2719
**Enhydrobacter**	0.343575	0.137929	0.236354	0.410525	0.3148	0.0630
**Vagococcus**	0.303999	0.000257	0.021087	0.479570	0.2279	0.0001
**Aquabacterium**	0.271143	0.029256	0.630718	0.049420	0.2225	0.1795
**Bacillus**	0.224723	0.155918	0.279896	0.191095	0.1749	0.0824
**Achromobacter**	0.195832	0.210806	0.259968	0.156611	0.2037	0.0020
**Geobacillus**	0.179772	0.036808	0.317472	0.095074	0.1622	0.0260
**Enterococcus**	0.173893	0.095200	0.058249	0.175211	0.1327	0.0007
**Delftia**	0.125895	0.556777	0.089860	0.148464	0.1222	0.0093
**Streptococcus**	0.086166	0.040675	0.013009	0.131607	0.0628	0.0136
**Dechloromonas**	0.084405	0.000272	0.199026	0.013706	0.0633	0.1158
**Cupriavidus**	0.084053	0.000045	0.200186	0.012349	0.0630	0.1914
**Holophaga**	0.077469	0.046445	0.100632	0.063331	0.0768	0.0686
**Legionella**	0.070932	0.074749	0.105686	0.049599	0.0721	0.0942
**Ralstonia**	0.069478	0.017309	0.155525	0.016473	0.0541	0.0003
**Pseudomonas_Escherichia**	0.046206	0.042759	0.075774	0.028130	0.0376	0.0389
**Comamonas**	0.034540	0.008443	0.064630	0.015961	0.0302	0.0356
**Kinneretia_Roseateles_ Pelomonas_Mitsuaria**	0.032672	0.000740	0.075733	0.006149	0.0251	0.0624
**Methyloversatilis**	0.026670	0.035222	0.029208	0.025107	0.0287	0.0441
**Aerococcus**	0.016152	0.004199	0.029415	0.007993	0.0146	0.0437
**Peredibacter**	0.015739	0.017823	0.015329	0.016063	0.0245	0.0295

MGInf: Midgut infected with trypanosomes; MGNInf: Midgut non-infected; HInf: Head infected; HNInf: Head non-infected; NT: Non teneral; T: Teneral
